# Intramuscular hemorrhages in the pathway of an electric current through the body — two case reports

**DOI:** 10.1007/s12024-022-00558-2

**Published:** 2022-11-08

**Authors:** Pawan Mittal, Michael Bohnert

**Affiliations:** 1 Department of Forensic Medicine, N.C. Medical College and Hospital, 132107 Panipat, Haryana India; 2 Institute of Forensic Medicine, University of Wuerzburg, Versbacher Str. 3, 97078 Würzburg, Germany

**Keywords:** Electrocution, Layered dissection, Intramuscular hemorrhage, Tetany

## Abstract

Intramuscular hemorrhages at autopsy can have a variety of traumatic as well as non-traumatic causes, but their recognition in electrical deaths is almost a rarity. We report on two autopsy cases of electrical fatalities, the first relating to a portion of the right upper human extremity, consisting (only) of the forearm and hand, while the other case relates to a female child who died after a high voltage electrical shock. In both cases, layered dissection of the upper limb revealed fresh intramuscular hemorrhages in the skeletal muscles that could be topographically related to the path taken by the current through the body. Externally visible electric marks were present in both cases. The hemorrhages were most likely caused by current-induced tetanic muscle contractions, producing an internal muscle trauma with rupture of fibers and bleedings. In complex situations, such as inconspicuous marks or a complete lack of visible signs on the body, the finding may be helpful in solving the case in consideration of the case history and circumstances. The vitality, topography, and pattern of the hemorrhages are discussed in the light of the available literature.

## Introduction

The diagnosis of a death by electrocution is mainly made on the basis of the external findings. The flow of electric current through the human body has specific effects on the excitable tissues, but morphological signs may be sparse or even absent [1]. The problem is further accentuated by the fact that there are no specific internal findings suggesting death by electrocution, especially in cases without any externally visible electric marks. In a few cases, however, one may occasionally find intramuscular hemorrhages which are produced by tetany-induced muscle contractions [2]. These hemorrhages are mostly seen in the skeletal muscles located in the current pathway, such as the upper limb and shoulder girdle muscles [3].

A unique case of suicide by electrocution committed by an electrician, who used coin electrodes fixed to his chest and a time switch, has been reported by Anders et al. [4]. During autopsy, a blackish linear mark was noticed on the parietal pleura of the left thoracic cavity topographically connecting the cutaneous current marks. Histologically, current- and heat-related changes, such as hypercontraction bands of the intercostal muscles and coagulative changes in the perineurium of peripheral nerves, were demonstrated. Anders et al. [5] also reported a case of suicide by electrocution in an electrical engineer who used a home-made device consisting of a connecting plug, scissors, and a magnifying glass. At autopsy, intramuscular hemorrhages were found in the skeletal muscles of the arms and the upper back. Based on the topographical distribution and microscopic pattern of the skeletal muscle alterations, the authors concluded that the hemorrhages were of vital origin and caused by current-induced tetanic muscle contractions.

Two more autopsy cases are hereby described, one relating to a right upper human limb while the other deals with a female child who died after sustaining a high-voltage electric shock. In each case, superficial and deep hemorrhages were seen in the skeletal muscles of the upper extremity that could be topographically associated with the current path in the body.

## Case reports

### Case no. 1

#### Brief history and details of the scene

**Fig. 1:**
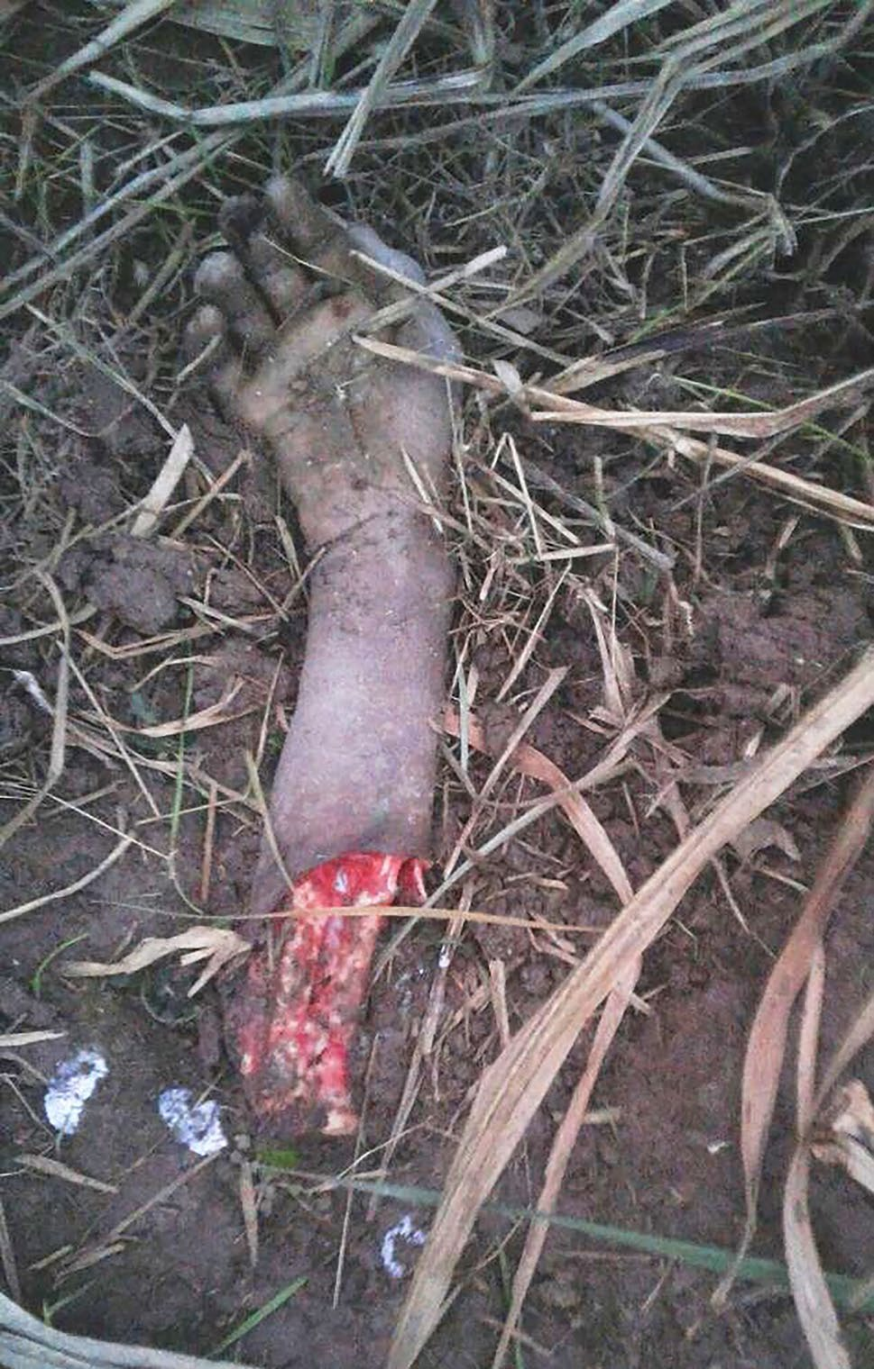
Human right upper limb at the scene. Soft-tissue defect on the forearm as well as dark blackish and yellowish regions are visible on the hand

The police brought part of a right upper human extremity for autopsy. The extremity consisted of the right forearm and the hand, apparently fresh and well articulated, found in supine position on a muddy surface, and surrounded by yellow plant twigs (Fig. 1). Purportedly, the limb was detected in a sugarcane field from where it was reported to the local police station by the owner of the field. The police arrived at the scene of discovery, took photographs, and conducted investigations. There was no source of electricity nearby and no other body parts were found in the surrounding area, even after a thorough search.

#### Autopsy findings

The limb was apparently fresh and showed no signs of putrefaction. It was smudged with muddy brownish stains at some places. Rigor mortis had passed off from the small joints of the hand. Faint bluish-purple postmortem lividity was present on the anterior aspect of the forearm. The skin was lax and wrinkled, hyperpigmented, atrophic, with complete loss of turgor. The forearm’s soft-tissue defect was circular in shape with pale, scalloped, and wrinkled margins, free of any ecchymoses, and smudged with foreign matter (i.e., of postmortem origin). The ulnar head was missing while its exposed region showed a zig-zag defect with adjacent tiny punctures typical of animal scavenging. No sharp injuries were present on the skin and bones.

**Fig. 2:**
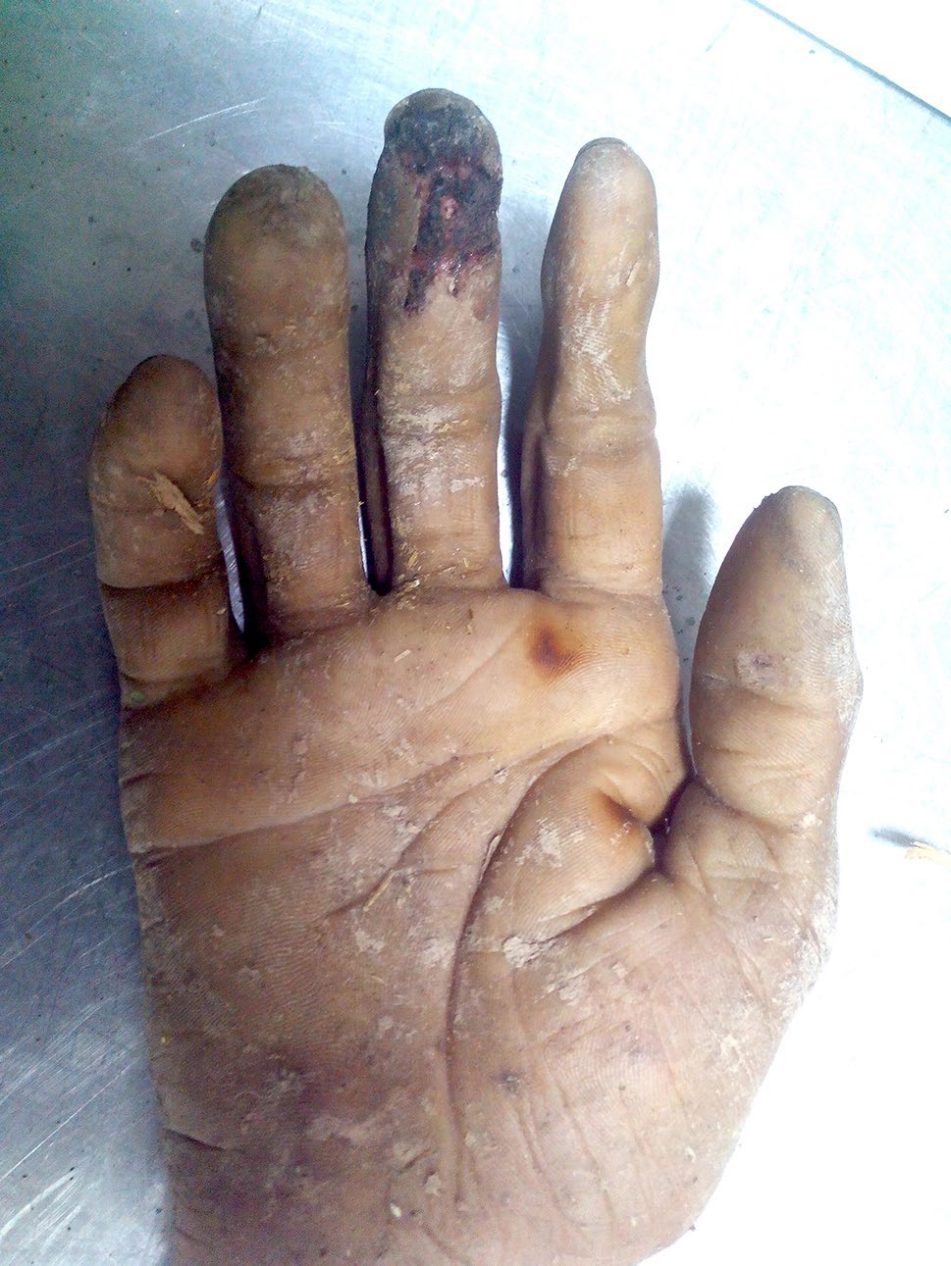
Right hand with (1) a typical charred lesion on the terminal phalanx of the middle finger, (2) a crater-like lesion on the thumb, and (3) two brownish ochre-yellow scorched lesions on the base of the right index finger and first web space. Bluish gray discoloration of the skin near the wrist and tip of the thumb

The following observations were made on the hand (Fig. 2):

1. A blackish charred area with a depressed and irregular surface on the terminal phalanx of the right middle finger that was still emitting a slight smell resembling burnt paper upon closer scrutiny. The margins of the wound showed signs of nibbling. On dissection, the underlying tissues were slightly congested with some petechial hemorrhages and smudged with clumps of dark grayish debris (suggesting metallic deposits). A similar lesion was present on the back of the proximal interphalangeal joint.
2. A wedge-shaped soft-tissue defect on the distal phalanx of the thumb near the interphalangeal joint. The defect showed a flattened, congested base and irregular margins. It was surrounded by prominent skin ridges.
3. There were two brownish-ochre areas of scorched skin, one between the base of the right index and middle finger and the other near the first web space.

The topography and appearance of the marks was consistent with electrical burns. In addition, confluent areas of bluish-gray discolorations were present at the thenar and hypothenar as well as the distal region of the right thumb, possibly due to metallization.

**Fig. 3:**
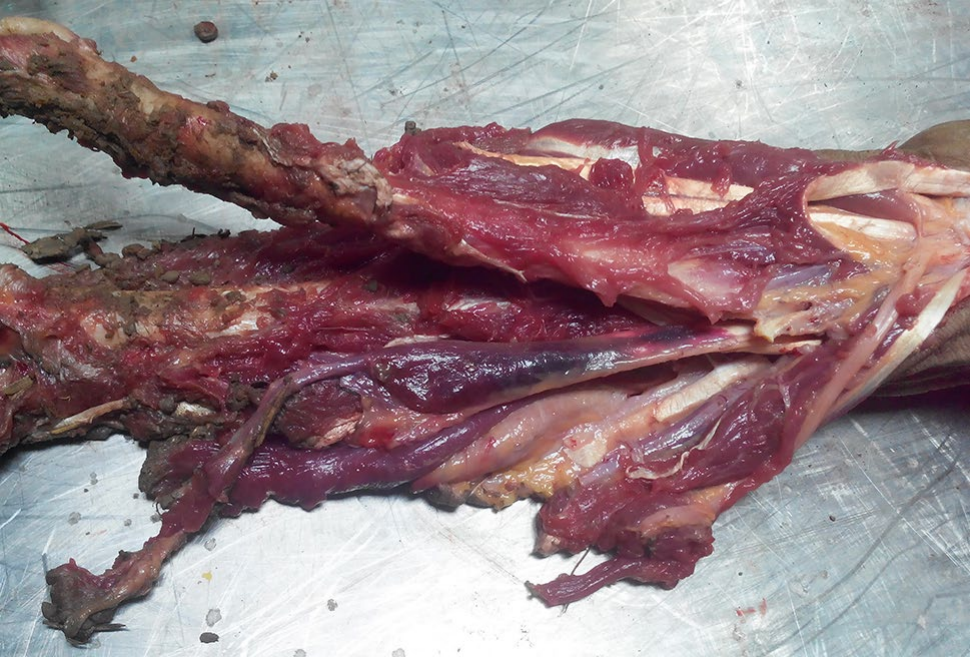
Intramuscular hemorrhages in the flexor muscle and tendon beneath an intact fascia and sheath, respectively (anatomically related to the right middle finger)

Complete circumferential and careful layered dissection of the forearm did not reveal any ecchymoses in the subcutis. At this stage, it was noticed that one of the flexor muscles and its tendon were diffusely hemorrhagic (Fig. 3). The overlying muscle fascia and tendon sheath were intact. The tendon was anatomically related to the right middle finger. Upon dissection, a hemorrhage was also seen in the depth of the muscle. The extensor compartment muscles of the forearm revealed similar superficial and also deep subfascial hemorrhages.

From anthropological evaluations and the appearance and texture of the soft tissue, the limb belonged to a middle-aged to old man with a height of about 175.1 ± 4.1 cm. A poorly visible greenish tattoo mark was merging with the forearm’s soft-tissue defect. The tattoo mark revealed a Hindu male’s name, written in Hindi (probably belonging to the decedent). Until today, this case has remained unsolved.

### Case no. 2

#### Brief history and details of the scene

This case deals with a 13-year-old girl who, along with her mother, was cutting grass on the side of a trail that was in the middle of waterlogged fields. According to her mother, she sustained an electric shock from a bare wire running over the bottom and side pole of a high-voltage transformer standing nearby. The child was declared “dead on arrival” in the hospital about 1 h after the incident. No resuscitation was carried out. The body was brought in for autopsy on the next day, about 23 h after the incident.

#### Autopsy findings

**Fig. 4:**
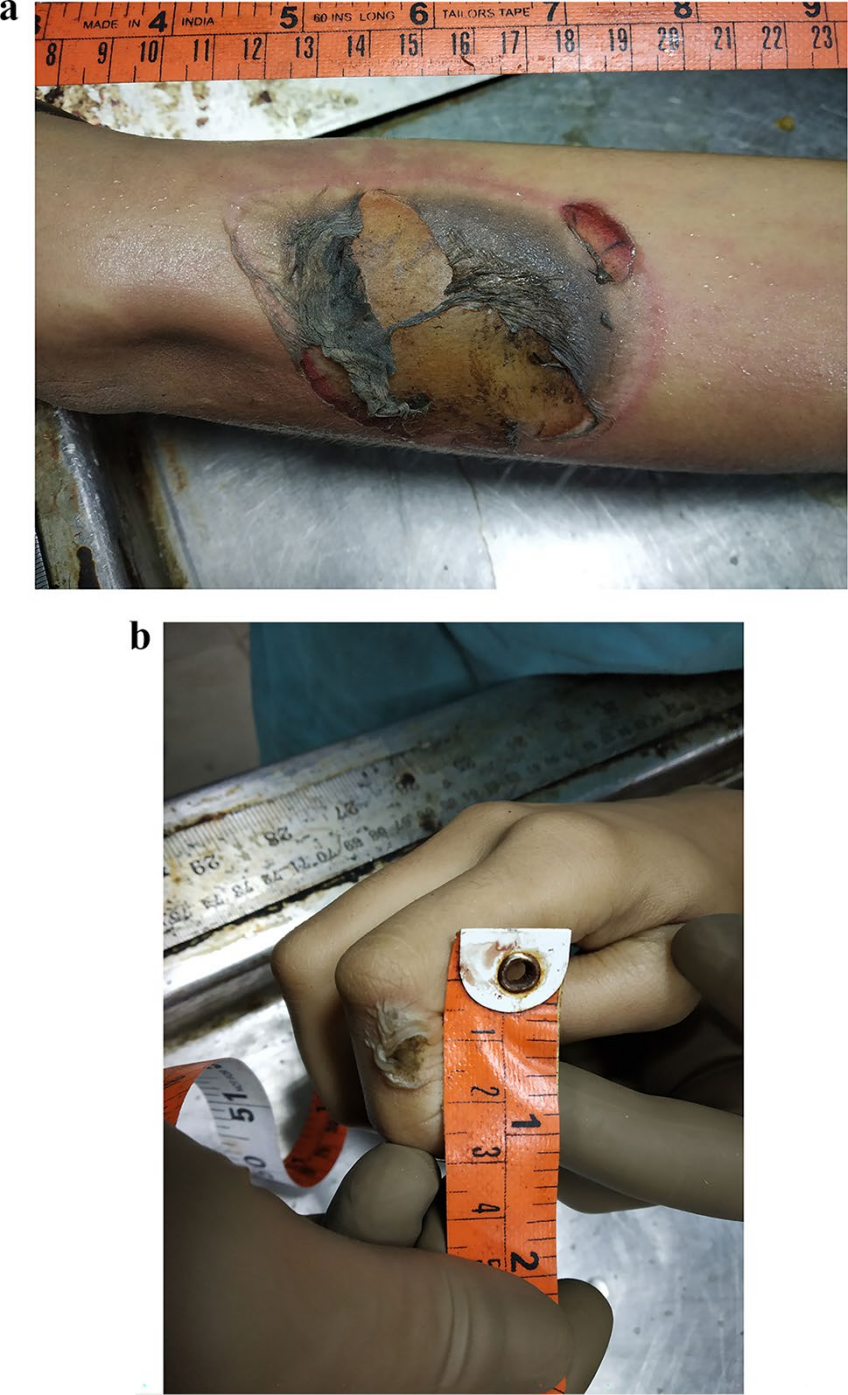
A typical target-like current mark with its three zones and blistering at the right forearm. A focal fern-like pattern is present superiorly. Inset: typical current entrance mark on the right index finger. The central area shows metallization surrounded by blistering, pallor, and hyperemia

The body was that of a female child of average build with a height of 142 cm and a body weight of 35.5 kg. The lower legs and feet were patchily smudged with mud stains. No conjunctival petechiae were present. No external injuries were visible except for the following electric marks:

1. Multiple current marks in the form of typical targetoid lesions (i.e., centrally flattened, charred, and metalized areas surrounded by zones of blistering, blanching, and hyperemia) were discernible on the posterior middle of the right index finger, the middle of the right arm, and the inferior aspect of the left forearm (Fig. 4).
2. A moderately sized contact electric burn was present, one on each side of the midline of the lower and anterior chest. The surface of each burn showed a fine net-like pattern of the white vest worn by the deceased. The right contact burn additionally showed spark burns in the vicinity. The overlying layers of clothes also revealed corresponding burn defects with curled up margins.

**Fig. 5:**
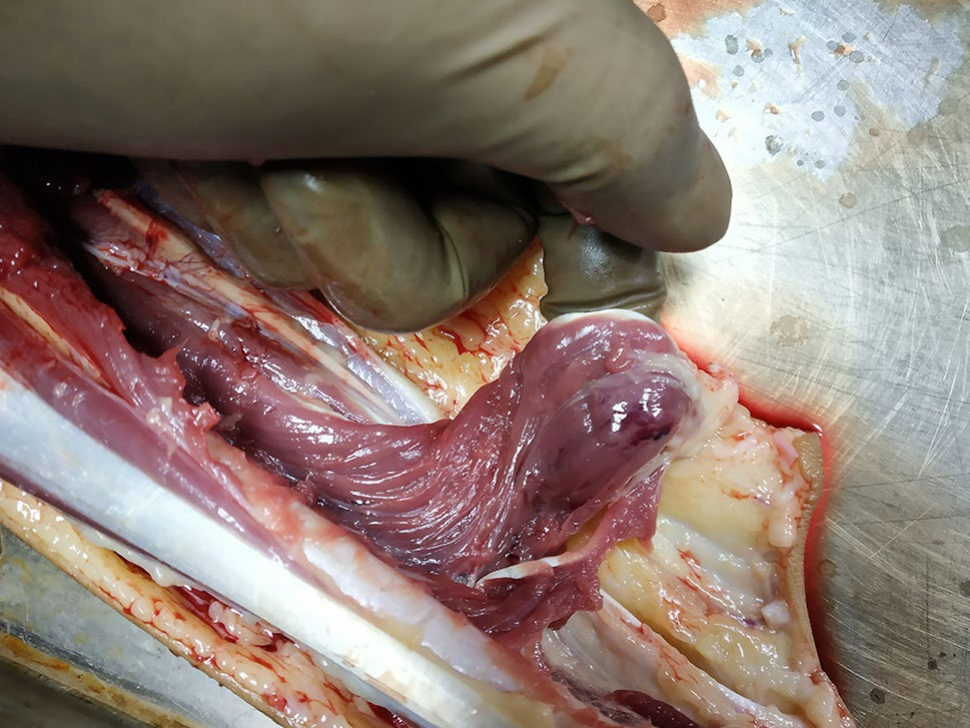
Flexor muscle of the right arm showing punctate to confluent subfascial hemorrhages

Layered dissection of the soft tissues of the right arm, the forearm, the shoulder girdle, and the upper back did not reveal any bleeding into the skin or subcutaneous fat. On further dissection, the flexor compartment muscles of the right arm and forearm showed punctate to confluent areas of bleeding beneath an intact fascia (Fig. 5). The bleedings displayed a flow-like pattern in some places. Similar areas of intramuscular hemorrhages were seen in the right deltoid and supraspinatus muscles.

The tracheal lumen contained abundant coarse whitish froth that could be traced to the segmental bronchi in diminishing quantity. Pulmonary emphysema and edema, along with Tardieu’s spots and occasional large patches of pleural ecchymoses, were seen. There was moderate cerebral edema with white matter petechiae, primarily focused on the thalamus and caudate nucleus. Some dotted subendocardial hemorrhages were present in the left ventricular papillary muscles and outflow tract. Pronounced generalized visceral congestion was observed. The cause of death was electrocution.

Due to the lack of technical possibilities, we were not able to perform histological examinations of the intramuscular hemorrhages or internal organs in both cases.

## Discussion

Both our cases showed intramuscular bleedings of the upper limbs with external electrocution marks. In both cases, there were no external signs of blunt injuries, and dissection did not show any bleedings in the subcutis. So, it was very likely that the bleedings were due to the electrical current.

Intramuscular hemorrhages may be the result of mechanical trauma, but also have been described in cases of drowning [6], hanging [7], hypothermia [8], electrocution [3, 4], and natural deaths from a cardiac or pulmonary cause [9]. The proposed mechanisms responsible for these hemorrhages are convulsive spasms during the asphyxiation process that cause hypercontraction, overexertion, and strain-induced rupture [6, 7]. In hypothermia-related deaths, systemic vasoconstriction, hypoxia-induced endothelial damage as well as mechanical vascular damage due to shivering have also been held responsible [8]. Tetany-induced muscle contractions are said to be responsible for rupture and bleeding into the muscle fibers in electrocutions as well [3]. These hemorrhages are described as tiny to moderately sized, confluent to strip-like bleedings, and are localized in the superficial and deep compartment muscles of the arms and forearms, the shoulder girdle, the upper back, and the intercostal muscles, thereby suggesting the path taken by the current through the body [3, 4]. However, any other external injury and any postmortem artificial bleeding have to be ruled out before proposing the lesions to be of electrical origin. This might be challenging in forensic casework.

Histological differentiation of vital (agonal) from postmortem (sustained during transportation and/or rough handling of the corpse, etc.) intramuscular hemorrhages has been evaluated in some studies, utilizing routine staining methods as well as immunohistochemistry [6, 7, 10, 11]. The findings suggestive of a vital nature of muscular hemorrhages are discoid and segmental disintegration of the muscle fibers, funnel-like concavities with empty and intact sarcolemmal tubes, and appearance of pathological longitudinal striation. A star-shaped or cobweb-like, centrifugally oriented bleeding pattern in the deep muscle fibers has been suggested to be helpful in differentiating a traumatic from a non-traumatic origin, as well as vital from postmortem bleedings [6, 7, 9]. During a retrospective analysis of 37 cases of fatal electrocutions, Karger et al. [1] provided a histological account of the intramuscular hemorrhages in selected cases. The authors found ruptures of fibers and moderate bleeding in the flexor muscles of the forearms along the current pathway.

However, the validity regarding the vitality of these alterations was doubted in another study [12]. Henssge et al. examined samples of skeletal muscle taken from 20 human corpses for estimating the time since death by looking for an idiomuscular bulge or tetanic contraction in the supravital period. Additional examination of the muscles by light microscopy revealed that the findings, previously interpreted as being of intravital origin, could also be produced post mortem [12]. The authors concluded that structural changes in the muscle fibers cannot be used as sole proof of vital mechanical or electrical traumatization and may also be produced by postmortem trauma, especially in the supravital period [12]. The non-validation of the proposed vital nature of the muscular hemorrhages/alterations in the agonal period is due to the fact that muscular tissue is excitable by a variety of mechanical, electrical as well as pharmacological stimuli for a prolonged time period in the (postmortem) supravital period (of cellular life), thereby being able to generate responses and alterations akin to vitality which are apparent on a gross as well as a microscopic level [12, 13]. So, as in other cases, histological examination may be helpful but not mandatory to distinguish vital from artificial findings.

Intramuscular hemorrhages as an internal sign of electrocution are a rarely reported finding. Anders et al. reported only two cases with intramuscular bleedings in a total of eight cases with a secured current path through the upper extremities during a period of 16 years [3]. An important problem in this context may be that layered dissection of the muscles, especially those of the limbs, is not always performed. These two case reports should indicate the need of this simple but helpful technique at autopsy of suspected electrocution deaths.

## Key points

1. Intramuscular hemorrhages at autopsy can have a wide variety of causes, which all require careful interpretation regarding their cause and vitality.
2. Tetanic muscle contractions triggered by the current flow can lead to hemorrhages into the skeletal muscles and/or tendons that may be demonstrated at autopsy.
3. These hemorrhages are topographically related to the current path through the body. The muscles frequently involved are the flexor–extensor groups of the upper limbs.
4. The histological changes may be helpful in indicating the vital nature of muscular lesions, but the findings do not provide absolute validity.
5. The presence of tetanic intramuscular hemorrhages, especially in the limb muscles, in the absence of visible current marks is an area still to be investigated.

## References

[1] Karger B, Süggeler O, Brinkmann B (2002). Electrocution — autopsy study with emphasis on “electrical petechiae”. Forensic Sci Int.

[2] Wick R, Byard RW, Tsokos M (2009). Electrocution and the autopsy. Forensic pathology reviews.

[3] Anders S, Tsokos M, Püschel K (2002). Pathways of electric current through the body. Rechtsmedizin.

[4] Anders S, Matschke J, Tsokos M (2001). Internal current mark in a case of suicide by electrocution. Am J Forensic Med Pathol.

[5] Anders S, Schulz F, Tsokos M (2000). Intramuscular haemorrhages of vital origin in a case of suicide by electrocution. Rechtsmedizin.

[6] Püschel K, Schulz F, Darrmann I, Tsokos M (1999). Macromorphology and histology of intramuscular hemorrhages in cases of drowning. Int J Legal Med.

[7] Schulz F, Buschmann CT, Braun C, Püschel K, Brinkmann B, Tsokos M (2011). Haemorrhages into the back and auxiliary breathing muscles after death by hanging. Int J Legal Med.

[8] Zátopková L, Hejna P, Palmiere C, Teresiński G, Janík M (2017). Hypothermia provokes hemorrhaging in various core muscle groups: how many of them could we have missed?. Int J Legal Med.

[9] Schulz F, Lach H, Püschel K, Tsokos M (2009). Nontraumatic intramuscular hemorrhages associated with death caused by internal diseases. Forensic pathology reviews.

[10] Fechner G, Bajanowski T, Brinkmann B (2005). Immunohistochemical alterations after muscle trauma. Int J Legal Med.

[11] Fechner G, Hauser R, Sépulchre M, Brinkmann B (2005). Immunohistochemical investigations to demonstrate vital direct traumatic damage of skeletal muscle. Int J Legal Med.

[12] Henssge C, Wang H, Hoppe B (2002). Light microscopical investigations on structural changes of skeletal muscle as artifacts after postmortem stimulation. Forensic Sci Int.

[13] Madea B (1994). Importance of supravitality in forensic medicine. Forensic Sci Int.

